# Supplementary material for: An empirical study for mitigating sustainable cloud computing challenges using ISM-ANN
Source: PLoS One. 2024 Sep 30;19(9):e0308971. doi: 10.1371/journal.pone.0308971 (PMC11441707; doi:10.1371/journal.pone.0308971)
Supplement: Attachment — Submitted filename: review-cloud-computing.pdf [file pone.0308971.s001.pdf]

## Unlocking the Future: A Groundbreaking Model for Mitigating Sustainable Cloud Computing Challenges through Empirical Studies

This paper empirically investigates the ethical challenges and practices of cloud computing about sustainable development. They conducted a systematic literature review followed by a questionnaire survey and identified 11 sustainable cloud computing challenges (SCCCs) and 66 practices for addressing the identified challenges. Interpretive structural modeling (ISM) and Artificial Neural Networks (ANN) were then used to identify and analyze the interrelationship between the SUCCs. Then, based on the results of the ISM, 11 process areas were identified to develop the proposed sustainable cloud computing challenges mitigation model (SCCCMM). The SCCCMM includes four main categories: Requirements specification, QoS and SLA, Complexity and Cyber security, and Trust. The model was subsequently tested with a real-world case study that was connected to the environment. In a sustainable cloud computing organization, the results demonstrate that the proposed SCCCMM aids in estimating the level of mitigation. The participants in the case study also appreciated the suggested SCCCMM for its practicality, user-friendliness, and overall usefulness. When it comes to the sustainability of their software products, we believe that organizations involved in cloud computing can benefit from the suggested SCCCMM. Additionally, researchers and industry practitioners can expect the proposed model to provide a strong foundation for developing new sustainable methods and tools for cloud computing.

The following are comments/suggestions to improve the manuscript:

1. Define symbols and abbreviations on their first use. Eg QoS, SUCCs and SLA.
2. Please include recommendation for developers and policy makers from your study
3. There are many typos. Please find and correct.
4. Please write in passive speech. Avoid pronouns we, their, our, them etc
5. The literature review is not sufficient. Include the following papers on neural networks and nature inspired algorithms:

    1. HUNTER: AI based holistic resource management for sustainable cloud computing, Journal of Systems and Software, Volume 184, 2022, 111124, ISSN 0164-1212, https://doi.org/10.1016/j.jss.2021.111124.
    2. Robust Tracking Control for Quadrotor UAV With External Disturbances and Uncertainties Using Neural Network Based MRAC, in IEEE Access, vol. 12, pp. 36183-36201, 2024, doi:10.1109/ACCESS.2024.3374894
    3. Genetic Algorithm Tuned Super Twisting Sliding Mode Controller for Suspension of Maglev Train With Flexible Track, in IEEE Access, vol. 11, pp. 30955-30969, 2023, doi: 10.1109/ACCESS.2023.3262416.
