# Supplementary material for: An empirical study for mitigating sustainable cloud computing challenges using ISM-ANN
Source: PLoS One. 2024 Sep 30;19(9):e0308971. doi: 10.1371/journal.pone.0308971 (PMC11441707; doi:10.1371/journal.pone.0308971)
Supplement: Attachment — Submitted filename: Response to Editor and Reviewer.docx [file pone.0308971.s002.docx]

Response to Editor and Reviewer’s Comments

PONE-D-24-13688

Unlocking the Future: A Groundbreaking Model for Mitigating Sustainable Cloud Computing Challenges through Empirical Studies

PLOS ONE

Dear Mohamed Rafik N. Qureshi, Ph.D.
Academic Editor
PLOS ONE

Many thanks for providing an opportunity to invite us to submit a revised version of the manuscript that addresses the points raised during the review process.

Editor Comments:

Comment 1: Please ensure that your manuscript meets PLOS ONE's style requirements, including those for file naming.

Response: Many thanks for your kind suggestions, we followed the PLOS ONE’s style requirements accordingly in the updated version of the manuscript.

Comment 2: You indicated that ethical approval was not necessary for your study. We understand that the framework for ethical oversight requirements for studies of this type may differ depending on the setting and we would appreciate some further clarification regarding your research. Could you please provide further details on why your study is exempt from the need for approval and confirmation from your institutional review board or research ethics committee (e.g., in the form of a letter or email correspondence) that ethics review was not necessary for this study? Please include a copy of the correspondence as an ""Other"" file.

Response: Many thanks for your kind suggestions, we upload an ethical approval copy of the correspondence as an “Other” file.

Comment 3: Please provide additional details regarding participant consent. In the ethics statement in the Methods and online submission information, please ensure that you have specified (1) whether consent was informed and (2) what type you obtained (for instance, written or verbal, and if verbal, how it was documented and witnessed). If your study included minors, state whether you obtained consent from parents or guardians. If the need for consent was waived by the ethics committee, please include this information.

Response: Many thanks for your kind consideration. Initially, during the survey we first sent a consent form to participants. Those show willingness in participation, we only sent the online survey link to them, to get feedback response from them. We sent a written consent form to them. We sent and obtained the consent form from the cloud computing experts.

Comment 4: In the online submission form, you indicated that [The data is available on request.]. All PLOS journals now require all data underlying the findings described in their manuscript to be freely available to other researchers, either 1. In a public repository, 2. Within the manuscript itself, or 3. Uploaded as supplementary information.
This policy applies to all data except where public deposition would breach compliance with the protocol approved by your research ethics board. If your data cannot be made publicly available for ethical or legal reasons (e.g., public availability would compromise patient privacy), please explain your reasons on resubmission and your exemption request will be escalated for approval.

Response: Many thanks for your kind suggestions, all the related data are presented it in detail in the manuscript. There is no other data, which are not mentioned in the submitted manuscript. We are agreeing to make our paper and its data publically available.

Additional Editor Comments:

The manuscript entitled 'Unlocking the Future: A Groundbreaking Model for Mitigating Sustainable Cloud Computing Challenges through Empirical Studies' needs further modification as per reviewers' comments.

Response: Many thanks for your kind appreciations, we modified our manuscript according to the reviewers’ comments.

**Reviewers' comments:**

Comment 1: Is the manuscript technically sound, and do the data support the conclusions?
The manuscript must describe a technically sound piece of scientific research with data that supports the conclusions. Experiments must have been conducted rigorously, with appropriate controls, replication, and sample sizes. The conclusions must be drawn appropriately based on the data presented.

Reviewer #1: Yes

Reviewer #2: Partly

Response: Many thanks for your kind appreciations. We again give full consideration with appropriate controls to recheck the tables data, sample sizes, replication and conclusion etc.

Comment 2: Has the statistical analysis been performed appropriately and rigorously?

Reviewer #1: Yes

Reviewer #2: No

Response: Many thanks for your kind appreciations. We proofread again the statistical analysis have been performed in the updated version of the manuscript.

Comment 3: Have the authors made all data underlying the findings in their manuscript fully available? The [PLOS Data policy](http://www.plosone.org/static/policies.action#sharing) requires authors to make all data underlying the findings described in their manuscript fully available without restriction, with rare exception (please refer to the Data Availability Statement in the manuscript PDF file). The data should be provided as part of the manuscript or its supporting information, or deposited to a public repository. For example, in addition to summary statistics, the data points behind means, medians and variance measures should be available. If there are restrictions on publicly sharing data—e.g. participant privacy or use of data from a third party—those must be specified.

Reviewer #1: Yes

Reviewer #2: No

Response: Many thanks for your kind appreciations. All the related data are presented it in detail in the manuscript. There is no other data, which are not mentioned in the submitted manuscript. We are agreeing to make our paper and its data publically available.

Comment 4: Is the manuscript presented in an intelligible fashion and written in standard English? PLOS ONE does not copyedit accepted manuscripts, so the language in submitted articles must be clear, correct, and unambiguous. Any typographical or grammatical errors should be corrected at revision, so please note any specific errors here.

Reviewer #1: Yes

Reviewer #2: Yes

Response: Many thanks for your kind appreciations.

Comment 6. PLOS authors have the option to publish the peer review history of their article ([what does this mean?](https://journals.plos.org/plosone/s/editorial-and-peer-review-process#loc-peer-review-history)). If published, this will include your full peer review and any attached files.

If you choose “no”, your identity will remain anonymous but your review may still be made public.

**Do you want your identity to be public for this peer review?** For information about this choice, including consent withdrawal, please see our [Privacy Policy](https://www.plos.org/privacy-policy).

Reviewer #1: No

Reviewer #2: No

Response: Many thanks for your kind suggestion. Kindly, don’t publish the peer review history.

Reviewer #1: Unlocking the Future: A Groundbreaking Model for Mitigating Sustainable Cloud
Computing Challenges through Empirical Studies

This paper empirically investigates the ethical challenges and practices of cloud computing about sustainable development. They conducted a systematic literature review followed by a questionnaire survey and identified 11 sustainable cloud computing challenges (SCCCs) and 66 practices for addressing the identified challenges. Interpretive structural modeling (ISM) and Artificial Neural Networks (ANN) were then used to identify and analyze the interrelationship between the SUCCs. Then, based on the results of the ISM, 11 process areas were identified to develop the proposed sustainable cloud computing challenges mitigation model (SCCCMM). The SCCCMM includes four main categories: Requirements specification, QoS and SLA, Complexity and Cyber security, and Trust. The model was subsequently tested with a real-world case study that was connected to the environment. In a sustainable cloud computing organization, the results demonstrate that the proposed SCCCMM aids in estimating the level of mitigation. The participants in the case study also appreciated the suggested SCCCMM for its practicality, user-friendliness, and overall usefulness. When it comes to the sustainability of their software products, we believe that organizations involved in cloud computing can benefit from the suggested SCCCMM. Additionally, researchers and industry practitioners can expect the proposed model to provide a strong foundation for developing new sustainable methods and tools for cloud computing.

Response: Many thanks for your kind appreciations.

Comment 1: Define symbols and abbreviations on their first use. Eg QoS, SUCCs and SLA.

Response: Many thanks for your kind suggestions for the improvement of the manuscript. We define the symbols and its abbreviations according in the updated version of the manuscript.

Comment 2: Please include recommendation for developers and policy makers from your study.

Response: Many thanks for your kind suggestions. We included recommendations for developers and policy makers in the Study Implication Section in the updated version of the manuscript.

Comment 3: There are many typos. Please find and correct.

Response: Many thanks for your kind suggestions. We proofread the updated version accordingly and correct the typos.

Comment 4: Please write in passive speech. Avoid pronouns we, their, our, them etc.

Response: Many thanks for your kind suggestions. We avoid the pronoun in the updated version accordingly.

Comment 5: The literature review is not sufficient. Include the following papers on neural networks and nature inspired algorithms:

1. HUNTER: AI based holistic resource management for sustainable cloud computing, Journal of Systems and Software, Volume 184, 2022, 111124, ISSN 0164-1212, <https://doi.org/10.1016/j.jss.2021.111124>.
2. Robust Tracking Control for Quadrotor UAV With External Disturbances and Uncertainties Using Neural Network Based MRAC, in IEEE Access, vol. 12, pp. 36183-36201, 2024, doi:10.1109/ACCESS.2024.3374894
3. Genetic Algorithm Tuned Super Twisting Sliding Mode Controller for Suspension of Maglev Train With Flexible Track, in IEEE Access, vol. 11, pp. 30955-30969, 2023, doi: 10.1109/ACCESS.2023.3262416.

Response: Many thanks for your kind suggestions for the improvement of the paper. We cited the mentioned the papers the updated version of the manuscript.

Reviewer #2: Title of the paper: Unlocking the Future: A Groundbreaking Model for Mitigating Sustainable Cloud Computing Challenges through Empirical Studies

The paper provides empirical analysis to identify 11 sustainable cloud computing challenges (SCCCs) and 66 practices for addressing the identified challenges. Interpretive structural modeling (ISM) and Artificial Neural Networks (ANN) were used to identify and analyze the interrelationship between the SUCCs. Then, based on the results of the ISM, 11 process areas were identified to develop the proposed sustainable cloud computing challenges mitigation model (SCCCMM). The SCCCMM includes four main categories: Requirements specification, QoS and SLA, Complexity and Cyber security, and Trust. The model was subsequently tested with a real-world case study that was connected to the environment.

Response: Many thanks for your kind appreciations.

Comment 1: Authors should justify the title through its novelty to justify the 'Groundbacking model'.

Response: Thanks for your kind consideration and suggestions.

This research justifies its title through novelty, and the authors demonstrate the following elements for novelty justifications:

- **Innovative Approach:** The authors showed that their model introduces a new methodology or technique that has not been previously explored in the field of sustainable cloud computing. This could involve a novel application of existing technologies, a new framework for analysis, or a unique combination of methods that offer significant improvements over existing solutions.
- **Empirical Validation:** The title mentions empirical studies, it implies that the model has been tested and validated through real-world data or experiments (case study conducted in Iflexion Company). The authors present clear and robust empirical evidence that supports the effectiveness of their model. This includes detailed descriptions of the study design, data collection, analysis methods, and a thorough discussion of the results.
- **Significant Impact on Sustainable Cloud Computing Challenges:** The model address specific challenges in sustainable cloud computing, such as energy consumption, carbon emissions, resource efficiency, or scalability. The paper clearly articulates these challenges and demonstrates how the proposed model provides effective solutions.
- **Relevance and Application:** The paper discusses the practical implications of the model, showing how it can be implemented in real-world cloud computing environments. This includes potential benefits for cloud service providers, users, and policymakers, as well as any limitations or considerations for deployment.
- **Contribution to the Field:** The authors position their work within the current body of knowledge, highlighting how their contributions advance the field. This includes discussing how the model builds on or diverges from existing research, its potential for future research, and its impact on the development of sustainable cloud computing practices.

By thoroughly addressing these points, the authors substantiate the claim of groundbreaking work and justify the significance of their research title, ensuring that it reflects the novelty and importance of their contributions to the field.

Comment 2: Please refer to 'The participants in the case study also appreciated the suggested SCCCMM for its practicality, user-friendliness, and overall usefulness. How this conclusion was derived.

Response: Thanks for your kind consideration and suggestions.

To derive the conclusion that "The participants in the case study also appreciated the suggested SCCCMM for its practicality, user-friendliness, and overall usefulness," the authors followed a structured approach in their research methodology. Here's how such a conclusion is typically reached in empirical studies:

- **Participants Selection:** The study involves selecting a group of participants who are representative of the target audience for the SCCCMM (Sustainable Cloud Computing Challenge Mitigation Model). These participants were professionals in the field of cloud computing, IT, sustainability experts, or users of cloud services.
- **Implementation and Testing:** The model implemented in a controlled setting, or the participants asked to apply it in their operational environment. This step is crucial for gathering firsthand data on the model's performance and its interaction with real-world variables.
- **Data Collection Methods:** The data collection methods such as surveys, interviews, or focus groups, employed to gather feedback from the participants. These instruments include questions or prompts designed to assess aspects of the model such as its practicality, user-friendliness, and overall usefulness.
- **Qualitative and Quantitative Analysis:** The responses and data collected were then analyzed. Qualitative data come from open-ended responses or interview transcripts that provide insights into the participants' thoughts and feelings about the model. Quantitative data involve ratings or structured responses that offer measurable feedback on various aspects of the model.
- **Synthesis and Conclusion Drawing:** The final step involves synthesizing all the data to draw conclusions. If a significant number of participants provide positive feedback on the model's practicality, user-friendliness, and usefulness, and the data show statistically significant satisfaction or performance improvement, we justifiably conclude that the model is appreciated by its users.
- **Reporting:** These findings and the methodologies used to derive was meticulously documented in the study, providing transparency and allowing others to replicate or build upon the research.

This process not only supports the validity of the conclusions drawn but also provides a comprehensive understanding of how the model performs in practical settings, directly reflecting the experiences and evaluations of its users.

Comment 3: Snowball sampling is a no-probability technique consist sample bias and margin of error hence authors should justify its use in the present study.

Response: Thanks for your kind consideration and suggestions.

Snowball sampling is a non-probability sampling technique often used in qualitative research, especially when the population is hard to reach or when the research focuses on a specific subset of people that are not easily accessible. Despite its limitations, such as potential sample bias and a non-quantifiable margin of error, there are circumstances where using snowball sampling can be justified:

- **Access to a Hidden or Difficult to reach Population:** Snowball sampling is particularly useful when the population of interest is rare, hidden, or otherwise difficult to access. For instance, studies involving highly specialized professionals, marginalized groups, or participants engaging in sensitive or illegal activities often rely on snowball sampling.
- **Building Trust:** In studies where trust is a significant barrier to participation, snowball sampling can be advantageous. It relies on social networks and introductions made by participants, which can help in gaining the trust of potential respondents, encouraging them to participate.
- **Initial Exploratory Phase:** Snowball sampling can be suitable in the exploratory phases of a research project, where the aim is to gather initial data and insights to develop a more rigorous, possibly quantitative, follow-up study.
- **Cost and Time Efficiency:** When resources are limited, snowball sampling can be a cost-effective and timely method for gathering data, especially compared to other methods that might require more extensive outreach and recruitment strategies.

To justify the use of snowball sampling in a study, we clearly articulate why this method is the most appropriate given the research objectives and the specific challenges associated with accessing the study population. We also discuss the steps taken to mitigate potential biases, such as attempting to reach diverse nodes within the network or cross-verifying information through additional data collection methods. Additionally, it was crucial for us to be transparent about the limitations of our sampling method when interpreting and generalizing findings from our study.

Comment 4: Please refer to ‘We emailed experts and networked with them on sites like LinkedIn, Facebook, and Research Gate. From July to August 2023,’ contradicts snowballing.

Response: Thanks for your kind consideration and suggestions.

The statement "We emailed experts and networked with them on sites like LinkedIn, Facebook, and ResearchGate. From July to August 2023" appears to contradict the principles of snowball sampling. Snowball sampling typically involves identifying initial subjects who then refer other participants, creating a chain of referrals. Here’s how we can justify their approach despite this apparent contradiction:

- **Combination of Methods:** We clarified that our study employed a combination of sampling methods. While we initiated contact with experts through direct emails and social networking sites, these initial contacts could then have referred additional participants, which align with the principles of snowball sampling.
- **Initial Contact Phase:** The direct emailing and networking might represent an initial phase to identify the initial seed participants. Once these key participants were engaged, snowball sampling techniques could have been used to expand the sample size by leveraging the networks of these initial contacts.
- **Snowball Sampling as a Secondary Method:** We argued that snowball sampling was used as a secondary method after establishing initial contacts. This hybrid approach allows for broad initial outreach while still leveraging the chain-referral mechanism inherent in snowball sampling to reach additional participants who might not be accessible through traditional methods.
- **Justification of Practically:** The practicality and feasibility of accessing experts directly through professional networks can be highlighted. Given the specialized nature of the study population (experts in a particular field), direct contact might have been necessary to initiate participation, with snowball sampling used subsequently to expand the sample.
- **Mitigating Bias:** To address potential sample bias, we discussed how they ensured diversity in the initial contacts and subsequent referrals. For example, reaching out to experts from various geographic locations, institutions, and areas of expertise can help in minimizing bias and ensuring a more representative sample.
- **Transparency and Limitations:** Being transparent about the mixed-methods approach and clearly stating the limitations of this strategy can also help in justifying the use of snowball sampling. We acknowledged the potential for sample bias and discuss how it might affect the study’s findings and conclusions.

By elaborating on these points, the authors can provide a coherent rationale for their sampling strategy, demonstrating that while direct outreach was used to initiate contact, snowball sampling principles were subsequently applied to expand the participant pool in a practical and effective manner.

Comment 5: MICMAC is used for classifying the variable/factor under study into four categories to draw interpretations from such variables. MICAMAC is not for detecting the challenges present or not in the given quadrant.

Response: Thanks for your kind consideration and suggestions.

MICMAC is a method used primarily for the classification of variables based on their influence and dependence within a system. It helps in understanding and interpreting the relationships among various factors in a study. Here are the key points regarding the use of MICMAC.

- **Classification of variables:** MICMAC classifies variables into four categories based on their levels of influence and dependence:

i. Autonomous Variables: Low influence and low dependence
ii. Dependent Variables: Low influence but high dependence
iii. Linkage Variables: High influence and high dependence
iv. Independent Variables: High influence but low dependence

- **Purpose and Classification:** The main purpose of MICMAC is to map out and analyze the structural interactions among variables. It is used in strategic planning and decision-making processes to identify critical factors that can significantly impact the system under study.
- **Not for Detecting Challenges:** MICMAC is not designed to detect whether specific challenges are present in the given quadrants. Instead, it helps in understanding how variables interact and the role they play within the system. This understanding aids in drawing interpretations and strategic insights rather than simply identifying challenges.

By focusing on the influence and dependence of variables, MICMAC helps researchers and decision-makers to prioritize actions, allocate resources efficiently, and anticipate the impact of changes within the system.

Comment 6: Please refer to "Figure 1: Research Methodology" What types of data were used for building ANN and ISM may be clarified. And how they were combined to provide SUCCMM.

Response: Thanks for your kind consideration and suggestions:

In the first step of this research a Systematic Literature Review (SLR) was conducted to identify sustainable cloud computing challenges (SCCCs) and its practices.

In the second step, we validate the findings of the SLR with the help of empirical study (online questionnaire survey).

In the 3^rd^ step of this research, we conducted an Interpretive Structural Modeling (ISM) approach for the categorization and finding relationships between the sustainable cloud computing challenges (SCCCs). When the relationship between two or more variables is complex, the ISM method can help you find it. The procedure followed by the ISM methodology to establish the link between SCCCs classification is depicted in Figure 2.

In the fourth step of this study, the ANN approach was used. 70% of the data in the current study is used to train the network model and Tenfold cross-validation is performed to ensure model validity while 30% of the data is used to test the model's network. The input-layer of the ANN model receives ten variables (SCCC1-10) as input. The output layer of the ANN model includes the dependent variable, sustainable cloud computing adoption, as an output.

We added the following details in the updated version of the manuscript for more clearification:

**Combining ANN and ISM for SUCCM**

1. **Data Collection:**

- **ANN Data:** Gathered through SLR and Empirical Study to create a robust dataset for training the neural network. This data is pre-processed and normalized to ensure consistency and accuracy.
- **ISM Data:** Collected via surveys, expert online interview, and focus groups to identify and understand the relationships between different sustainability factors in cloud computing.

2. **Model Building:**

- **Training ANN:** The collected quantitative data is used to train the ANN model. The ANN learns patterns and dependencies within the data, enabling it to predict outcomes and optimize various cloud computing parameters.
- **Constructing ISM:** Constructing the qualitative data, ISM is applied to establish a hierarchical structure of the factors influencing sustainable cloud computing. This involves identifying key variables and mapping their direct and indirect relationships.

3. **Integration:**

- **Hybrid Framework:** The outputs of ANN and ISM are integrated into a comprehensive framework. The ANN provides predictive insights and optimization strategies, while the ISM offers a structured understanding of the interdependencies among factors.
- **Model Validation:** The integrated model is validated using additional data sets and expert reviews to ensure its accuracy and reliability in mitigating cloud computing challenges.
- **Implementation:** The validated SUCCMM is then implemented, combining the predictive power of ANN with the strategic insights from ISM to provide a holistic approach to sustainable cloud computing.

The methodology involves collecting both quantitative data for ANN and qualitative data for ISM, using these models to address different aspects of the problem. ANN handles data-driven optimization, while ISM structures the problem contextually. Together, they form SUCCMM, a comprehensive model to tackle sustainable cloud computing challenges effectively.

Comment 7: LOTEC (Lyapunov Optimization on Time and Energy Cost) may be Lyapunov Optimization on Time and Energy Cost ( LOTEC).

Response: Many thanks for your kind suggestion. We corrected it according in the updated manuscript.

Comment 8: All responses were recorded using a 5-point Likert scale, authors may provide a questionnaire and data file.

Response: Many thanks for your kind suggestions. The paper size is almost 38 pages. We will provide the questionnaire survey data file as supplementary file.

Comment 9: Please refer to 'Survey data were analyzed in this study using frequency analysis [55]' analysed results like pilot testing, reliability, validity and other statistical results may also be included in the manuscript.

Response: Many thanks for your kind consideration and suggestions. Yes these types of analysis can also be apply on our data and may be added in the manuscript. But due to paper size, we cannot add it in this manuscript. In future, we will added in the extended version of this paper all types of test like pilot testing, reliability, validity and other statistical results.

Comment 10: Please refer to 'Based on industry feedback, we created the SSIM as depicted in Table I.' Authors may provide more clarity on how the process of industry feedback was registered.

Response: Many thanks for your kind suggestions. We added the following details with Table 1 discussion in the updated manuscript:

## Collecting Industry Feedback for SSIM

## Identify Key Industry Experts:

- **Criteria for Selection:** We selected experts based on their experience, expertise in sustainable cloud computing, and their role in the industry. Aim for diversity in terms of sectors (e.g., technology providers, sustainability consultants, academics).
- **Recruitment Process:** We reach out to potential participants through professional networks, industry associations, and direct invitations.

## Data Collection Methods:

- **Surveys:** We developed structured surveys with questions designed to gather insights on the importance and influence of various factors in sustainable cloud computing. We Use Likert scales, multiple-choice questions, and open-ended questions to collect both quantitative and qualitative data.
- **Interviews:** We conducted semi-structured interviews with selected experts to obtain in-depth qualitative data. These interviews can provide nuanced understanding and context that surveys alone might not capture.
- **Focus Group:** We organized focus groups where experts discuss and debate the interrelationships between factors. These sessions can help in validating and refining the initial findings from surveys and interviews.

## Evaluating Industry Feedback for SSIM:

**Data Analysis**

- **Qualitative Analysis:** We perform thematic analysis on the qualitative data obtained from interviews and open-ended survey responses. Identify common themes, patterns, and insights regarding the relationships between factors.
- **Quantitative Analysis:** We analyze the quantitative survey data using statistical methods such as frequency analysis, to identify the most critical factors and their perceived influence.

**Constructing the SSIM**

- **Develop Initial SSIM:** Based on the analyzed feedback, we construct an initial version of the SSIM by mapping out the relationships between factors. Each cell in the matrix indicates the direct influence of one factor on another.

**Validation and Refinement:** We validate the initial SSIM through additional rounds of feedback. This can be done by sharing the matrix with a broader group of experts or through follow-up workshops and focus groups. Make necessary adjustments based on the feedback received.

Comment 11: How the accuracy of the ANN model is ensured using limited data.

Response: Many thanks for your kind consideration. Ensuring the accuracy of an Artificial Neural Network (ANN) model with limited data involves using a combination of strategies that maximize the utility of the available data while preventing overfitting and improving generalization. Cross-validation particularly k-fold cross-validation, involves splitting the data into k subsets and training the model k times, each time using a different subset as the validation set and the remaining data as the training set. In this paper, we applied the Cross-validation k-fold as our data were too limited.

Comment 12:  Authors may refer to the following references for ISM, MICAMC and its validation:

Qureshi, K.M., Mewada, B.G., Alghamdi, S.Y., Almakayeel, N., Mansour, M. and Qureshi, M.R.N., 2022. Exploring the lean implementation barriers in small and medium-sized enterprises using interpretive structure modeling and interpretive ranking process. Applied System Innovation, 5(4), p.84.
Qureshi, K.M., Mewada, B.G., Alghamdi, S.Y., Almakayeel, N., Qureshi, M.R.N. and Mansour, M., 2022. Accomplishing sustainability in manufacturing system for small and medium-sized enterprises (SMEs) through lean implementation. Sustainability, 14(15), p.9732.
Qureshi, M.N., Kumar, D. and Kumar, P., 2008. An integrated model to identify and classify the key criteria and their role in the assessment of 3PL services providers. Asia Pacific Journal of Marketing and Logistics, 20(2), pp.227-249.
Qureshi, M.N., Kumar, D. and Kumar, P., 2007. Modeling the logistics outsourcing relationship variables to enhance shippers' productivity and competitiveness in logistical supply chain. International Journal of Productivity and Performance Management, 56(8), pp.689-714.
Talib, F., Rahman, Z. and Qureshi, M.N., 2011. An interpretive structural modelling approach for modelling the practices of total quality management in service sector. International Journal of Modelling in Operations Management, 1(3), pp.223-250.

Response: Many thanks for your kind suggestion. We cited the mentioned references for ISM, MICMAC and its validation.

Dr. Anwar Ghani,

Corresponding Author
