# Supplementary material for: An empirical study for mitigating sustainable cloud computing challenges using ISM-ANN
Source: PLoS One. 2024 Sep 30;19(9):e0308971. doi: 10.1371/journal.pone.0308971 (PMC11441707; doi:10.1371/journal.pone.0308971)
Supplement: Attachment — Submitted filename: V2 Response to Editor and Reviewers.docx [file pone.0308971.s003.docx]

Response to Editor and Reviewer's Comments

PONE-D-24-13688R1

Unlocking the Future: A Groundbreaking Model for Mitigating Sustainable Cloud Computing Challenges through Empirical Studies

PLOS ONE

Dear Mohamed Rafik N. Qureshi, Ph.D.
Academic Editor
PLOS ONE

Many thanks for providing an opportunity to invite us to submit a revised version of the manuscript that addresses the points raised during the review process.

Additional Editor Comments:

The manuscript entitled "Unlocking the Future: A Groundbreaking Model for Mitigating Sustainable Cloud Computing Challenges through Empirical Studies" may further be modified as per the old and new comments from both reviewers.

Author's Response: Many thanks for your kind suggestions. We modified the updated version according to the old and new comments from both reviewers.

Reviewers Comments to the Authors:

Comment 1: Is the manuscript technically sound, and do the data support the conclusions?

The manuscript must describe a technically sound piece of scientific research with data that supports the conclusions. Experiments must have been conducted rigorously, with appropriate controls, replication, and sample sizes. The conclusions must be drawn appropriately based on the data presented.

Reviewer #1: Partly

Reviewer #2: Partly

Author's Response: Many thanks for your kind appreciation:

- **Technically Sound Scientific Research:** This research adheres to established scientific principles and methods. It is well-designed, with clear objectives and methodologies that others can replicate in the field.
- **Data Supporting Conclusions:** The manuscript presents data that directly supports the conclusions drawn by the researchers. This data is robust, accurately collected, and analyzed using appropriate statistical methods.
- **Rigorous Experimentation:** Experiments were conducted rigorously, meaning they were carefully planned and executed to minimize biases and errors.
- **Appropriate Drawing of Conclusions:** Conclusions were drawn from the data based solely on the results obtained and logically followed from the findings presented. It was not extrapolated beyond what the data can support, nor should they ignore contradictory evidence.

In summary, the statement outlines the expectations for a high-quality scientific manuscript, emphasizing the importance of rigorous experimentation, robust data analysis, and the careful drawing of conclusions based on the evidence presented. These principles were followed as they are fundamental to maintaining the integrity and reliability of scientific research.

Comment 3. Has the statistical analysis been performed appropriately and rigorously?

Reviewer #1: No

Reviewer #2: Yes

Author's Response: Many thanks for your kind consideration.

To assess whether statistical analysis has been performed appropriately and rigorously, several key considerations were followed in our research that were addressed:

- **Methodological Clarity:** We followed ISM-ANN statistical methodologies.
- **Data Quality and Preprocessing:** The data is cleaned and prepared appropriately before analysis. We checked it for outliers and missing data and ensured that data transformation was justified and correctly applied.
- **Choice of Statistical Tests:** We choose appropriate statistical methods for the research question and type of data collected.
- **Sample Size and Power Analysis:** The sample size is justified based on power analysis or other considerations.
- **Interpretation of Results:** The results were interpreted appropriately in the context of the study's objectives. We discussed the implications of the findings and any limitations or assumptions made during the analysis.

Comment 4. Have the authors made all data underlying the findings in their manuscript fully available?

The [PLOS Data policy](http://www.plosone.org/static/policies.action#sharing) requires authors to make all data underlying the findings described in their manuscript fully available without restriction, with rare exception (please refer to the Data Availability Statement in the manuscript PDF file). The data should be provided as part of the manuscript or its supporting information, or deposited to a public repository. For example, in addition to summary statistics, the data points behind means, medians and variance measures should be available. If there are restrictions on publicly sharing data—e.g. participant privacy or use of data from a third party—those must be specified.

Reviewer #1: Yes

Reviewer #2: Yes

Author's Response: Many thanks for your kind appreciation.

5. Is the manuscript presented in an intelligible fashion and written in standard English?

PLOS ONE does not copyedit accepted manuscripts, so the language in submitted articles must be clear, correct, and unambiguous. Any typographical or grammatical errors should be corrected at revision, so please note any specific errors here.

Reviewer #1: No

Reviewer #2: Yes

Author's Response: Many thanks for your kind suggestions and appreciation. We previously proofread the manuscript. Once again, the updated version was thoroughly double-reviewed in order to reduce any grammatical mistakes and try our best to look like in Standard English.

**Reviewer #1:** My concerns are not addressed yet.
The literatures are not sufficient and not updated in this revised version too:

Author's Response: Many thanks for your kind suggestions and appreciation. We have already added the following mentioned papers in the updated version:

1. HUNTER: AI based holistic resource management for sustainable cloud computing, Journal of Systems and Software, Volume 184, 2022, 111124, ISSN 0164-1212, <https://doi.org/10.1016/j.jss.2021.111124>.
2. Robust Tracking Control for Quadrotor UAV With External Disturbances and Uncertainties Using Neural Network Based MRAC, in IEEE Access, vol. 12, pp. 36183-36201, 2024, doi:10.1109/ACCESS.2024.3374894
3. Genetic Algorithm Tuned Super Twisting Sliding Mode Controller for Suspension of Maglev Train With Flexible Track, in IEEE Access, vol. 11, pp. 30955-30969, 2023, doi: 10.1109/ACCESS.2023.3262416.

Now, we also added the following relevant literature in the 2^nd^ updated version of our manuscript:

Furthermore, since it eliminates excessive duplication in common subsystems such as collective data, computing and communication networks, and so on, an integrated system is more effective and environmentally friendly [20]. In addition, since it increases energy awareness, the integration contributes to a greener system [21].

Cloud services have added more data centers quickly, and this situation has resulted in environmental issues. Research by [22] shows that as the world's data consumption and processing needs continue to rise, so does the energy consumption and consequent carbon footprint of data centers. Carbon emission by clouds has become a significant concern because of its impact on global warming; therefore, there is research on the use of clean energy sources and options for carbon neutralization [23].

Resource management is significant for cutting down the impact of cloud computing services on the natural environment. Shreshth et al. [15] argue that effective energy management should focus on achieving high server occupancy, VM placement and workload scheduling to achieve lower energy usage with high resource productivity. Thus, the goals mentioned above can be attained effectively by means of dynamic provisioning and workload consolidation, for instance [24].

When implementing sustainable solutions, cloud providers face another problem – the security and privacy of the data. This paper draws from the literature [25] and reveals the appropriate encryption techniques, access control models, and data protection laws. As for the second research question, research has been conducted to find out how contactless solutions, particularly utilizing hybrid cloud models and secure multi-party computation techniques, have been used in the attempt to increase security while at the same time not losing sight of the gains that could be attained in terms of sustainability.

Thus, the real problem is to guarantee clear economic efficiency and applicability of sustainable cloud solutions for the broad scope of digitalization. Research [26] focuses on ways of adopting renewable sources of energy for business, ways to reduce operating expenses and how benefits from green accreditation can be harnessed to appeal to consumers. Risk management is also a part of business continuity planning that aims to reduce any form of disruption that may occur due to the impacts of environmental factors or changes in the set-apart regulations.

Therefore, the use of effective governance frameworks and the right regulatory policies are important in enhancing sustainability in cloud computing businesses. International practices and support for green technologies (European Commission, 2021; U. S. Department of Energy, [27] assesses the norms, laws, and policies of the countries stress on environmental legislations for green technology implementation. Coordinated efforts by government, business, and academic circles are critical to the enhanced generation of sustainable cloud computing agendas across the world.

**Reviewer #2:** 1. The manuscript has several typos; hence, it needs careful reading. Professional English editing is recommended.

Author's Response: Many thanks for your kind suggestions and appreciation. We previously proofread the manuscript. Once again, the updated version was thoroughly double-reviewed in order to reduce any grammatical mistakes and try our best to look like in Standard English.

Comment 2. Please refer to the previous query, "Authors should justify the title through its novelty to justify the 'Groundbacking Model'." There are several papers published on the ISM-ANN methodology. The methodology used does not provide novelty; hence, the title should be suitably modified.

Author's Response: Many thanks for your kind suggestions and appreciation. We modified the title accordingly to "An Empirical Study for Mitigating Sustainable Cloud Computing Challenges Using ISM-ANN."

Comment 3. The title "Unlocking the Future: A Groundbreaking Model for Mitigating Sustainable Cloud Computing Challenges through Empirical Studies" may be modified as "An Empirical Study for Mitigating Sustainable Cloud Computing Challenges Using ISM-ANN." or on a similar line.

Author's Response: Many thanks for your kind suggestions. We modified the title accordingly.

Dr. Anwar Ghani

Corresponding author.
